# First Report of Biochemical Mechanisms of Insecticide Resistance in the Field Population of *Culex pipiens* (Diptera: Culicidae) from Sari, Mazandaran, North of Iran

**Published:** 2019-12-31

**Authors:** Seyed Hassan Nikookar, Mahmoud Fazeli-Dinan, Seyyed Payman Ziapour, Fatemeh Ghorbani, Yaser Salim-Abadi, Hassan Vatandoost, Ahmad Ali Hanafi-Bojd, Ahmad Ali Enayati

**Affiliations:** 1 Department of Medical Entomology and Vector Control, School of Public Health and Health Sciences Research Center, Addiction Institute, Mazandaran University of Medical Sciences, Sari, Iran; 2 Department of Medical Entomology and Vector Control, School of Public Health and Health Sciences Research Center, Mazandaran University of Medical Science, Sari, Iran; 3 Department of Parasitology, Zoonosis Research Center, Pasteur Institute of Iran, Amol, Iran; 4 Department of Medical Entomology and Vector Control, School of Public Health, Tehran University of Medical Sciences, Tehran, Iran; 5 Department of Health Services and Health Promotion, School of Health, Rafsanjan University of Medical Sciences, Rafsanjan, Iran; 6 Department of Chemical Pollutants and Pesticides, Institute for Environmental Research, Tehran University of Medical Sciences, Tehran, Iran

**Keywords:** *Culex pipiens*, Insecticide resistance, Enzyme, Iran

## Abstract

**Background:**

*Culex pipiens* play an important role in transmission of infectious diseases. Vector control by chemical pesticides, leads inevitably to resistance development. Understanding the underlying resistance mechanisms can help improve the control programmes and insecticide resistance management.

**Methods:**

The total contents of cytochrome p450s and the activities of glutathione S-transferases, alpha- and beta-esterases and inhibition rates of acetylcholine esterase (by propoxur) were measured in the field population of *Cx. pipiens* collected from Sari County, North of Iran, in 2016 and the results were compared with those of the laboratory susceptible strain according to the biochemical assay methods of WHO for adult mosquitoes. Independent sample *t*-test was used to compare the mean values of enzyme activities/contents between filed and laboratory susceptible populations.

**Results:**

The enzyme ratio of cytochrome p450s, alpha- and beta-esterases in the field population was 2.07, 3.72 and 1.36 respectively when compared with the results of the laboratory population. Although not statistically significant, the mean GSTs activities in the field population was marginally less than the laboratory population (ER=0.92). Acetylcholinesterase was insensitive to propoxur in 62.82% of the individuals of the tested field population. There was a significant difference (P< 0.05) between all values of the activities/contents of the enzyme in the field population except for GSTs compared with the laboratory susceptible strain. The highest enzyme activity was related to alpha esterase.

**Conclusion:**

The present study showed a range of metabolic mechanisms, comprising p450s and esterases combined with target site insensitivity of AChE, contributing to organophosphate, carbamate and pyrethroid resistance in the field population of *Cx. pipiens*.

## Introduction

Mosquitoes are the most important arthropods of medical importance transmitting various diseases to humans and animals. Among mosquitoes, the genus *Culex*, especially, *Cx. pipiens* complex are known vectors of several human pathogens including West Nile virus, St. Louis encephalitis virus, Rift Valley fever virus, Japanese encephalitis virus, *Wuchereria bancrofti* (1–3) as well as zoonotic pathogens including dog heartworm (4) and avian malaria (5). Mosquito-borne diseases immensely affect public health and cause problems in terms of economy and development all over the world (6).

*Culex pipiens* is widely distributed in almost all continents throughout the world (7, 8). Lately, *Cx*. *pipiens* has been arrived on Newfoundland’s Avalon Peninsula, Canada and southern Sweden (9, 10). It shows a vast geographical distribution in most parts of Iran (11–13). The distribution, dominance and high abundance of *Cx*. *pipiens* have been reported from northern Iran (14–18). The use of chemical insecticide is one of the most widely practiced strategies for mosquito-borne diseases control (19). Given the important role of pesticides in mosquito control programms, the development of resistance to insecticides is of great concern for diseases control (20, 21), recently led to many outbreaks of mosquito-borne diseases (22). Resistance to insecticides in *Cx. pipiens* has been reported from many parts of the world (23, 24).

In Iran, approximately 14,000 tons of active ingredient agricultural pesticides were imported or produced (25). Population and environmental studies have alarmingly revealed high levels of pesticide residues in the environment (25), it could possibly be a cause of concern for development of resistance to pesticide in mosquito populations that have a water-related life cycle.

*Culex pipiens* was resistant to DDT, propoxur, lambda-cyhalothrin, cyfluthrin and deltamethrin (26–29), tolerant to deltamethrin (27, 28) and susceptible to malathion (28). In a recent study, however, revealed relatively high resistance to propoxur, malathion, fenitrothion and dieldrin in the field populations of *Cx. pipiens* from Sari County, North of Iran (30).

The two basic mechanisms of insecticide resistance in mosquitoes are metabolic resistance and target site insensitivity. Metabolic resistance is mainly caused by elevated levels of enzyme activities that lead to detoxification or sequestration of insecticides before they reach to their target site (31). Glutathione S-transferases (GSTs), cytochrome p450s and esterase’s (ESTs) are three main enzyme groups responsible for metabolic resistance to the main classes of insecticide applied against insects of public health importance (32, 33). Elevated level of activeties of these enzymes was shown to be responsible for resistance to pesticides in a variety of mosquitoes including *Anopheles stephensi* (34, 35), *Aedes aegypti* (36, 37) and *An. culicifacies* (38). Biochemical studies showed the involvement of cytochrome p450s in the metabolic resistance to four major insecticide classes in *An*. *funestus*, *An*. *stephensi* and *An. darlingi* (39–41). Increased levels of esterases have been associated with resistance to organophosphates, carbamates and pyrethroids in *Cx*. *quinquefasciatus* and *Helicoverpa armigera* (42). Involvement of glutathione S-transferases has been observed in resistance to organophosphates, organochlorines and pyrethroid insecticides in *An. subpictus*, *Cx. pipiens* and *Rhipicephalus bursa*, *Musca domestica*, *Drosophila melanogaster* and *Nilaparvata lugens* (43–45). Glutathione S-trasferases are principally responsible for resistance to organochlorine insecticides and their involvement in resistance to other groups of insecticides is secondary (44). There is also evidence for involvement of the insensitive acetylcholinesterase (AChE) in resistance to organophosphates and carbamates in *Cx*. *pipiens*, *Ae*. *aegypti* and *Ae*. *albopictus* (46, 47).

Esterases showed more important role in the detoxification of organophosphate insecticides used against *Cx. pipiens* in Egypt (48). DDT, organophosphate and pyrethroid resistance was characterized in *Cx. pipiens* from Turkey and cytochrome p450s and esterases were involved in resistance (21). Overproduced esterases and insensitive AChE were involved in enzymatic resistance of the field populations of *Cx*. *pipiens* from Grand Tunis area, Northeast Tunisia (49). Other resistance mechanisms, including target-site resistance mutations, can be involved in insecticide resistance in *Cx. pipiens* populations from the Mediterranean region (50, 51). The kdr mutation (L1014F) has been reported in association with resistance to lambda-cyhalothrin from *Cx. pipiens* in Morocco region (51). The L1014S mutation has been observed in *Cx. pipiens* pallens from Japan and China. The L1014C and V1016G mutations have only been reported in *Cx*. *pipiens* form *molestus* from China and Saudi Arabia, respectively (23). The kdr mutation along with elevated levels of p450s and GSTs, are associated with pyrethroid resistance in the populations of *Cx. pipiens* from China (52). Different amplified esterases (encoded by the Ester super locus) and two substitutions on the AChE1 (encoded by the ace-1gene) including the G119S and the F290V substitutions have been reported to confer resistance to a great variety of organophosphate and carbamate insecticides in *Cx. pipiens* (50). The G119S mutation is common in *Cx. pipiens* populations in Europe, Africa and China but F290V mutation does not have a large dispersal in *Cx. pipiens* populations outside the Mediterranean region (53).

*Culex pipiens* is a dominant species in Iran (11), especially Mazandaran Province (14, 15, 18). It is an important vector of the West Nile Virus in the world (1). Evidence of the West Nile virus circulating in the north of the country (54) and the presence of wetlands for migrating birds (reservoir hosts), is a cause for concern for the spread of the virus in the province. In a recent study in Mazandaran Province, resistance to several insecticides has been documented in *Cx. pipiens* (30), however, there is no report on the mechanisms of insecticide resistance in this species from Iran. Therefore, determination of the underlying insecticide resistance mechanisms of *Cx. pipiens* are important for better insecticide resistance management and implementation of effective control programs if need be.

## Materials and Methods

### Study areas and sample collection

The present study was undertaken in Sari Township, the capital of Mazandaran Province, northern Iran, in 2016 with geographical coordinates of 36^°^33′48.70″ N and 53^°^03′36.35 E. It is situated between the Caspian Sea in the north and the Alborz mountain ranges in the south, Miandorud Township in the east and Qaemshahr Township in the west. The study area has a population of approximately 296,417 according to the 2011 census with an average annual temperature of 15 °C and precipitation of 789.2mm. It included two distinct geographical areas of mountainous and plain/littoral. Larvae collection was carried out using the dipping sampling method from artificial habitats (drainage channels) in the village of Qajar Kheil located in a plain area of Sari Township, in summer 2015. Ecologically, the sampling site had features including stagnant water, with depth of 50cm, expanse of 5m, muddy floor, without plant and shadow–sun conditions. Water temperature and average pH were 20 °C and 7.12, respectively. The main occupations of the residents of the sampling area are agriculture, horticulture, animal husbandry, fishing and handcrafts Diazinon, chlorpyrifos, imidacloprid, cypermethrin and glyphosate were the most commonly used pesticides by farmers in agriculture and animal husbandry in plain areas of Sari Township especially Ghajar kheil.

### Rearing and preparation of samples

Larvae were reared in standard insectary conditions 28±2 °C and 70±5% relative humidity in a 12: 12 dark: light photoperiod. Live unfed 2–3d old adult females were put in the −80 °C freezer for conducting biochemical assays. A susceptible laboratory strain of *Cx. pipiens* provided by the Department of Medical Entomology and Vector Control, School of Public Health, Tehran University of Medical Sciences, Iran was used for comparison.

### Biochemical assays

Activity levels of glutathione S-transferases (GSTs), alpha- and beta- esterases, and inhibition rates of acetylcholine esterase (by propoxur), contents of cytochrome p450s and total protein for adult mosquito specimens were measured according to (45, 55, 56) with minor modifications. Buffer solutions including 0.625M potassium phosphate pH 7.2, 0.025M sodium acetate pH 5, 1% triton sodium phosphate pH 7.8, 0.1M sodium phosphate pH 7, 0.02M sodium phosphate pH 7.2, 5% sodium dodecyl sulphate (SDS) diluted in 0.1M sodium phosphate pH 7, 0.1M sodium phosphate 6.5 and 50mM sodium phosphate pH 7.4 were prepared fresh and used for a maximum of two weeks. The rest of the solutions and reagents were made fresh during the tests being done. Forty-five deep-frozen female adult mosquitoes (45 specimens of the field and 45 susceptible) were individually put in wells of a 96-well microtiter plate (Maxwell®, China), initially, 100μl of cold dwater was added to each well and the specimens were homogenized by a steel pestle on ice. Then another 250μl of cold dwater was added and the plate was centrifuged at 1100g (3000rpm) in a refrigerated centrifuge (Beckman Coulter®, Inc., California, USA) at 4 °C for 20min. The supernatants were used as enzyme source for biochemical assays.

Absorbance levels were measured spectrophotometrically with a microplate reader (ELX808 Ultra Microplate Reader BIOTEK® Inc., California, USA) activated by KC-Junior software, at specific wavelengths for each enzyme. The mean absorbance was calculated based on data for the two replicate wells per mosquito. Three blank replicates were considered in all enzyme assays that include all reagents and working solutions related to activities of each enzyme except the enzyme source (instead of which distilled water was used).

### Cytochrome p450s assay

In a fresh microtiter plate, reaction mixture in each well consisting of 20 microliters of the homogenate in duplicate, 80 microliters of 0.625M potassium phosphate buffer pH 7.2, 200 microliters of 3,3′,5,5′ tetramethylbenzidine (TMBZ) solution (0.01g TMBZ dissolved in 5ml methanol plus 15ml of 0.25M sodium acetate buffer pH 5.0) and 25 microliters of 3% hydrogen peroxide were mixed. Then, plates were kept at room temperature for 2h and absorbance was read at 450nm as an endpoint. The enzyme contents were described as equivalent units of cytochrome (EUC) p450s /mg protein amended for the known heme content of cytochrome C and p450s using a standard curve of purified cytochrome C.

### Acetylcholinesterase (AChE) assay

Twenty-five microliters of homogenates were put in each well in duplicates followed by adding 145μl of Triton phosphate buffer (1% Triton X-100 in 0.1M phosphate buffer pH 7.8) to each replicate to solubilize AChE. Ten μl of DTNB solution (0.01M dithiobis-2-nitrobenzoic acid in 0.1M phosphate buffer pH 7.0) and 25μl of the substrate ASCHI (0.01M acetylthiocholine iodide) were added to one replicate to initiate the reaction. The latter solution was replaced by 25μl of the substrate ASCHI containing 0.2% of the inhibitor propoxur (0.1M) for the second test replicates. The kinetics of the enzyme reaction was monitored continuously at 405nm for 5 min. The percentages of inhibition of AChE activity by propoxur in the test were calculated relative to the uninhibited wells. The assay conditions were predetermined so that individuals without an insensitive AChE-based resistance mechanism had >60% inhibition of the AChE activity.

### General esterase assay

The alpha- and beta-naphthyl acetate substrates were used to measure the general esterases activity. Reaction mixtures included 20 microliters of the homogenate in duplicate (for each of the alpha- and beta esterases) in adjacent microtiter plate wells (assigned alpha and beta) and 200 microliters of alpha- and beta-naphthyl acetate solution (120μl of 30mM alpha- or beta-naphthyl acetate dissolved in 12ml 0.02M phosphate buffer pH 7.2) respectively. After 30min incubation period at room temperature, 50 microliters of fast blue solution (0.023g fast blue dissolved in 2.25ml distilled water and 5.25ml of 5% SDS 0.1M sodium phosphate buffer pH 7) was added to each well. After another incubation period at room temperature for 5min, the absorbance was measured at 570nm as an endpoint. The resulting optical densities (OD) were converted to product concentration using standard curves of ODs for known concentrations of the products alpha- and beta-naphthol, respectively. The enzyme activity was stated as μM of product formed/min/mg protein.

### GSTs assay

The reaction mixture contains 10μl of the homogenate in duplicate plus 200μl of working solution (10mM reduced glutathione dissolved in 0.1M phosphate buffer pH 6.5 and 3mM CDNB originally dissolved in methanol). The absorbance was kinetically read at 340nm for 5min. The enzyme activity was reported as mM of conjugate produced/min/mg protein using the extinction coefficient of CDNB corrected for the path length of the solution in the microtiter plate well.

### Protein assay

Protein content of mosquito homogenates was measured using Bradford method by adding 300 microliters of Bio-Rad reagent (prepared as 1: 4 dilution in ddH2O) to 10 microliters of supernatant in duplicates. The absorbance was measured at 570nm after the mixture was incubated for 5min at room temperature. Absorbance was converted into protein concentration using a bovine serum albumin standard curve obtained with the same method and reagents.

### Analysis of biochemical assays data

Raw data of readings from the plate reader were transformed into enzyme activities/ contents using the Microsoft Excel version 10 and the equations for each enzyme group. Independent-sample *t*-test using SPSS version 19 software (IBM, USA) was employed to compare the mean values of enzyme activities or contents between filed and laboratory susceptible populations. Enzyme ratios (ER) were computed by dividing the mean activities or content of different enzyme groups of the field strain with those of the laboratory susceptible strain. The P<0.05 was considered as statistically significant. The percentage inhibition of AChE by propoxur compared with non-inhibited reaction was computed and the threshold of >60% was considered as AChE insensitive to propoxur.

## Results

Our previous study on *Cx. pipiens* revealed that this species is highly resistant to all four major classes of insecticides including organochlorine, organophosphate, carbamate and pyrethroids, but the resistance level was lower to pyrethroids compared to other insecticides. The highest and the least mortality rates were produced by etofenprox and propoxur with a rate of 76.47% and 4.25%, respectively.

The mean and standard deviation of cytochrome p450s contents in the field and laboratory susceptible populations were 49×10^−6^± 299×10^−7^ and 282×10^−7^±235×10^−7^EUC cytochrome p450s/mg protein, respectively. The ratio of cytochrome p450s in the field population was 2.07 when compared with that of the susceptible laboratory population (Table 1). The differences between the contents of p450 in the field and laboratory susceptible populations were statistically significant (P= 0.04).

**Table 1.** Mean enzyme activity/content and enzyme ratio (ER) of MFO, GST, Alpha and Beta esterases in the field and susceptible laboratory populations of *Cx. pipiens*, summer 2016

**Enzymes**	**Population**	**Mean**	**ER**
**MFO**	Field Population	49 × 10^−6^	2.07
	laboratory susceptible Population	282 × 10^−7^	1
		
**GST**	Field Population	0.119	0.92
	laboratory susceptible Population	0.129	1
		
**Alpha**	Field Population	878 × 10^−6^	3.72
	laboratory susceptible Population	236 × 10^−6^	1
		
**Beta**	Field Population	1 × 10^−3^	1.36
	laboratory susceptible Population	7 × 10^−4^	1

The mean GST activity in the field population was marginally less than that in the laboratory susceptible population (0.119vs 0.129 mM/min/mg protein) with a ratio of 0.92 (Table 1). However, the difference of GSTs activity in the field and laboratory susceptible populations was statistically not significant (P> 0.05) at 5% level.

The mean activity of alpha- and beta-naphthyl acetate was 878×10^−6^ and 1×10^−3^μM/min/mg protein in the field population and 236× 10^−6^ and 7×10^−4^μM/min/mg protein in the laboratory susceptible population, respectively. The enzyme ratios in the field population were 3.72 and 1.36 for alpha- and beta-esterases when compared with those of the laboratory susceptible population (Table 1). There was a significant difference between the field and laboratory susceptible populations regarding the activity of alpha- (P< 0.001) and beta-esterases (P= 0.001).

Acetylcholinesterase biochemical assay showed that 62.82 % of the tested field population had insensitive AChE to propoxur as the inhibition of AChE was less than the threshold of 60% (Table 2).

**Table 2.** Inhibition of AChE activity by propoxur in the filed population of *Cx. pipiens* collected in summer 2016

**No of tested *Cx. pipiens***	**Inhibition rates of AChE**	**No of tested *Cx. pipiens***	**Inhibition rates of AChE**	**No of tested *Cx. pipiens***	**Inhibition rates of AChE**	**No of tested *Cx. pipiens***	**Inhibition rates of AChE**
**1**	74.92	21	65.8	41	36.5^*^	61	45.8^*^
**2**	52.3^*^	22	25.4^*^	42	86.7	62	60.4^*^
**3**	71.3	23	63.1	43	52.8^*^	63	53.1^*^
**4**	59.4^*^	24	41^*^	44	54^*^	64	54.9^*^
**5**	54.8^*^	25	13.4^*^	45	60.5^*^	65	71.2
**6**	45.7^*^	26	68.8	46	17.3^*^	66	57^*^
**7**	52.8^*^	27	36.6^*^	47	67	67	56.8^*^
**8**	38.1^*^	28	57.7^*^	48	59.9^*^	68	56.2^*^
**9**	73.1	29	58.5^*^	49	86.5	69	60.3^*^
**10**	76.76	30	38.5^*^	50	60.5^*^	70	82.5
**11**	68.4	31	78.1	51	65.3	71	65.6
**12**	73.4	32	49.6^*^	52	24.4^*^	72	51.9^*^
**13**	52.5^*^	33	37.2^*^	53	43.7^*^	73	65.2
**14**	63.5	34	76	54	50.3^*^	74	43.1^*^
**15**	32.3^*^	35	46.8^*^	55	21.7^*^	75	21.4^*^
**16**	76.3	36	64.7	56	59.2^*^	76	84.2
**17**	68.7	37	67.4	57	33.4^*^	77	16.8^*^
**18**	48.6^*^	38	59.5^*^	58	35.2^*^	78	64
**19**	84.3	39	75.9	59	10.8^*^		
**20**	66.4	40	2.6^*^	60	45.7^*^		

* The inhabitation rate is lower than the threshold of 60%

**Fig. 1.**
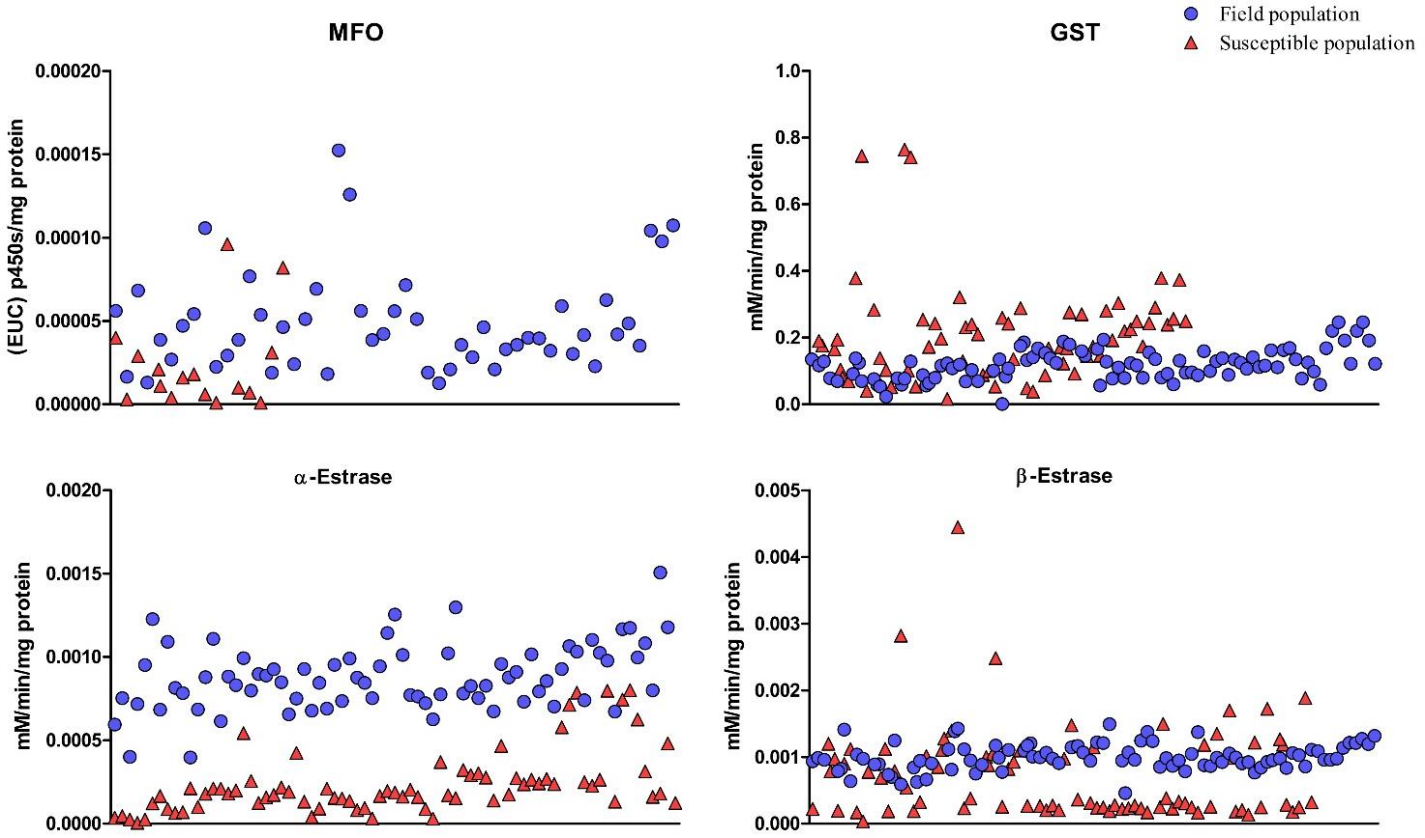
Scatter diagram of distribution of enzyme activities/contents levels in each individual in the field and laboratory susceptible populations of *Cx. pipiens* from Sari, Iran in 2016

Scatter diagram shows variations in enzyme levels in each individual in field and laboratory populations (Fig. 1).

## Discussion

*Culex pipiens* is considered to be a competent vector of pathogens in the world (1). This species is one of the most common mosquito species in Iran (11) and has a widespread distribution in the North of the country in the Caspian Sea region (14, 15).

In a recent study in the area *Cx. pipiens* showed resistance to organochlorine, organophosphorus, carbamates and pyrethroid insecticides (30). Development of insecticide resistance can be caused by direct selection pressure from insecticides used in vector control programmes and or indirectly by the use of agrochemicals (57). In this follow up study, elevated enzyme activities of alpha- and beta- esterases, cytochrome P450s and insensitive AChE in the filed population of *Cx. pipiens* confirmed the occurrence of resistance and also elucidated its underlying resistance mechanisms.

The results of this study indicated that different enzyme groups may play a role in insecticide resistance in the field population of *Cx. pipiens*. Alpha- and beta-esterases and cytochrome p450s levels were significantly higher in the filed population of *Cx. pipiens* compared to the laboratory susceptible population. Involvement of esterases and cytochrome p450s in DDT, malathion and pyrethroid resistance was described in populations of *Cx. pipiens* from Turkey (21). Zayed et al. showed an association between elevated levels of esterases activity in *Cx. pipiens* larvae with organophosphorus resistance (43) which is in accordance with the findings of our research.

Enhancements of cytochrome p450s in pyrethroid resistance were reported in *Cx. pipiens quinquefasciatus* (58), *Cx. pipiens pallens* (59), *An. stephensi* (32), *Ae. aegypti* and *Ae. albopictus* (60, 61). General esterases were mostly reported to be involved in resistance to organophosphates and carbamates, elevated levels of these enzymes have also been associated with resistance to organophosphates in *Cx. pipiens quinquefasciatus* (58), *Anopheles stephensi* (35) and cross resistance to pyrethroids (34).

In the present study, significant increase in alpha-esterases activity was discovered in the field population compared with other enzyme groups relative to the laboratory susceptible population. This higher level of enzyme activity can possibly suggest the significance of development of organophosphorus resistance in the field populations of *Cx. pipiens*. The development of resistance to insecticide in the study area could possibly be due to the heavy use of pesticides. Mazandaran is the leading province in terms of pesticide use based on its toxic load in the Iran (62). In recent years farmers have converted fields not previously cultivated into horticulture, and this is probably one reason for the high use of pesticides for pest control in the area Pesticides applied in agriculture can pollute streams running off farms and this could be the indirect path of how these insecticides may end up in larval habitats of mosquitoes. Consequently high usage of insecticides in horticulture and agriculture practices leads to emergence of resistance to insecticides in mosquito populations in many areas (63).

Similar observations have also been made in populations of *Cx. quinquefasciatus* from Malaysia and India (58, 64) and *Ae. aegypti* and *Ae. albopictus* from Thailand (47) associated with elevated alpha-esterases levels.

In this study the difference in the activities of glutathione S-transferases in susceptible laboratory and field populations were not statistically significant from each other. This is observed in similar studies in Iran (29). GSTs are mainly involved in organochlorine insecticides resistance (44). Whereas p450s and esterases are contributing to resistance to most groups of insecticides. As the population tested in the current study was highly resistant to many different organophosphorous (OP) and carbamates insecticides, this is quite natural, according to the economics of resistance, that the insects over-produce those enzymes with higher role in detoxifying more insecticide groups.

Acetylcholinesterase (AChE) is a key enzyme in the nervous system, hydrolyzing acetylcholine neurotransmitter and terminating neural impulses, and are the target for both organophosphates and carbamate insecticides, two main classes of pesticides applied for pest and vector control in agriculture and public health (22). In the present study, inhibition rates of AChE by propoxur showed the frequency of insensitive AChE gene in the filed population of *Cx. pipiens* to be higher than the threshold of <60% which confirmed its involvement in the resistance to organophosphates and carbamates. High insecticide resistance caused by insensitivity or reduced sensitivity in response to OPs and carbamate insecticides was observed in *An. gambiae*, *An. albimanus*, *Cx. vishnui*, *Cx. pipiens*, and *Cx. quinquefasciatus* (22). High insensitivity of acetylcholinesterase can be mainly due to mutations in the ace-1gene (22). Thus, molecular studies are recommended to verify the presence of these mutations in ace-1 gene in the field populations of *Cx. pipiens* in Mazandaran Province.

## Conclusion

The findings of this research provide the first evidence on the involvement of metabolic mechanisms including alpha- and beta-esterases, mixed-function oxidases and acetylcholinesterase in insecticide resistance in the field population of *Cx. pipiens* from Sari, Mazandaran Province, Iran*.* There is evidence of detection of WNV in the mosquito populations and records of human infection with the virus in North and North West of Iran. The findings of the present study provide valuable information on the underlying resistance mechanisms of this species which is pivotal in implementing successful control program of this important vector.
